# Intrinsic microRNA regulatory programs define lineage-specific differentiation in human mesenchymal stem cells of different origin – dental pulp- and fat tissue-derived

**DOI:** 10.1007/s12015-026-11107-7

**Published:** 2026-03-22

**Authors:** Carla Cristina G. Pinheiro, Camila M. Lopes-Ramos, Taro Inagaki, Paula Fontes Asprino, José Ricardo M. Ferreira, Ygor Gonçalves Félix de Mattos, Helena Coutinho Geiger Campos, Jamil Award Shibli, Raphael B. Parmigiani, Reza Jarrahy, Akishige Hokugo, Alessandra V. S. Faria, Daniela Franco Bueno

**Affiliations:** 1 Instituto de Ensino e Pesquisa, Hospital Sírio-Libanês, São Paulo, SP Brazil; 2 Channing Division of Network Medicine, Department of Medicine, Brigham and Women’s Hospital, Harvard Medical School, Boston, MA USA; 3 Regenerative Bioengineering and Repair Laboratory, Division of Plastic and Reconstructive Surgery, Department of Surgery, David Geffen School of Medicine at UCLA, Los Angeles, CA USA; 4 Department of Oral Medicine, Infection and Immunity, Division of Periodontology, Harvard School of Dental Medicine, Boston, MA USA; 5 RCrio Company, Campinas, São Paulo, Brazil; 6 Departamento de Biologia Celular e do Desenvolvimento, Universidade de Sao Paulo Instituto de Ciencias Biomedicas, São Paulo, SP Brazil; 7 School of Dentistry, Faculdade Israelita de Ciências da Saúde Albert Einstein, Hospital Israelita Albert Einstein, São Paulo, 05521-200 SP Brazil

**Keywords:** MicroRNA, Osteogenic differentiation, Mesenchymal stem cell, Dental pulp stem cell, Processed lipoaspirate cells

## Abstract

Cell fate determination and terminal differentiation are shaped by intrinsic molecular programs that coordinate lineage-specific gene expression. In mesenchymal stem cells (MSCs), variability in osteogenic efficiency among distinct tissue sources remains poorly understood and cannot be fully explained by differentiation conditions alone. Increasing evidence indicates that post-transcriptional regulatory mechanisms, particularly those mediated by microRNAs (miRNAs), are closely associated with differentiation outcomes. Here, we investigated whether intrinsic miRNA expression landscapes are associated with lineage-associated differences in late-stage mineralization, in the context of potential donor-related variability of human MSCs. Dental pulp stem cells (DPSCs) and processed lipoaspirate (PLA)-derived MSCs were cultured under identical osteogenic induction conditions and analyzed longitudinally throughout differentiation. Both cell populations fulfilled established mesenchymal phenotypic criteria and successfully initiated osteogenic commitment. However, DPSCs exhibited significantly enhanced extracellular matrix mineralization at mid-to-late stages of differentiation, suggesting divergence at later stages of extracellular matrix mineralization rather than at early lineage commitment. Temporal small RNA sequencing performed at days 0, 7, 14, and 21 revealed progressive remodeling of miRNA expression in both MSC sources. Direct comparison at the mature osteoblast stage identified a discrete set of ten miRNAs differentially expressed between DPSCs and PLA-derived cells. DPSCs displayed reduced expression of several miRNAs previously associated with inhibitory roles in osteogenic pathways. Network-based analyses indicated convergence of these miRNAs on gene programs involved in skeletal development and extracellular matrix organization. Targeted validation confirmed decreased expression of miR-10a-5p and miR-196a-5p, accompanied by increased expression of osteogenesis-associated genes, including BMP1 and MMP16, in DPSCs. Collectively, these findings show that lineage-specific miRNA expression patterns are associated with distinct osteogenic maturation trajectories in human MSCs. This work identifies an intrinsic post-transcriptional signature through which cellular origin may influence terminal differentiation outcomes and suggests that such miRNA profiles may serve as informative molecular biomarkers in the future for the selection and characterization of mesenchymal stem cells intended for human bone tissue engineering applications.

**Supplementary Information:**

The online version contains supplementary material available at 10.1007/s12015-026-11107-7.

## Introduction

Mesenchymal stem cells (MSCs) are widely recognized for their capacity for self-renewal, multipotent differentiation, and secretion of trophic and immunomodulatory factors that collectively contribute to tissue homeostasis and repair [1, 2]. At the cellular level, these properties reflect tightly regulated molecular networks that are involved in lineage commitment, differentiation progression, and maintenance of cellular identity. In the context of skeletal biology, MSCs serve as the principal progenitor population supporting osteoblast formation and bone remodeling. However, despite the clinical application of standardized differentiation protocols, substantial variability in osteogenic outcomes persists among MSC populations derived from different tissues or donors, representing a major limitation to reproducible differentiation and predictable biological responses [3].

Among the most extensively studied MSC sources, adipose-derived stem cells and dental pulp stem cells (DPSCs) have emerged as accessible and biologically relevant populations for investigating mechanisms associated with osteogenic differentiation [4, 5]. Adipose tissue obtained through liposuction yields the stromal vascular fraction, commonly referred to as processed lipoaspirate (PLA), which contains multipotent MSCs capable of differentiating along multiple mesenchymal lineages [6]. DPSCs, isolated from the dental pulp of deciduous or permanent teeth, exhibit high proliferative capacity and pronounced osteogenic and dentinogenic potential, supported by relatively standardized isolation protocols that promote population reproducibility [5]. Notwithstanding their shared mesenchymal phenotype, these cell types frequently display divergent differentiation efficiencies when subjected to identical osteogenic induction conditions, indicating that phenotypic equivalence does not necessarily reflect functional or regulatory equivalence [3, 7].

Dental pulp stem cells (DPSCs) represent an accessible and biologically relevant MSC population for studies of skeletal and craniofacial differentiation. Although bone marrow–derived mesenchymal stem cells (BMSCs) are considered a classical reference population for osteogenic differentiation, DPSCs can be obtained through minimally invasive procedures from exfoliated deciduous or extracted permanent teeth. Importantly, DPSCs originate from cranial neural crest cells, which play a central role in craniofacial morphogenesis, including the formation of bone and dental tissues. This developmental origin supports their use as a model for investigating osteogenic differentiation and extracellular matrix mineralization. Previous comparative studies have demonstrated that DPSCs exhibit osteogenic differentiation capacities comparable to or exceeding those of BMSCs under standardized induction conditions, further supporting their biological relevance for skeletal research.

Accumulating evidence indicates that MSCs retain lineage-specific molecular signatures associated with their tissue of origin, which are linked to differentiation propensity and lineage bias [3, 7]. These intrinsic features influence how cells interpret and respond to external differentiation cues, suggesting that osteogenic outcomes are not solely dictated by inductive conditions but may also be shaped by pre-existing molecular architectures. Consequently, reliance on osteogenic media alone may be insufficient to overcome inherent differences in differentiation competence. Defining the intrinsic factors associated with lineage-specific differentiation capacity is therefore essential for understanding heterogeneity within MSC populations and for elucidating fundamental aspects of cell fate regulation.

MicroRNAs (miRNAs) have emerged as important components of post-transcriptional gene regulatory networks that coordinate stem cell behavior, including proliferation, differentiation, apoptosis, and maintenance of stemness [7]. By modulating mRNA stability and translation, miRNAs contribute to the modulation of gene network dynamics rather than acting as binary on–off switches. During osteogenic differentiation, miRNAs have been shown to influence key signaling pathways involving RUNX2, bone morphogenetic proteins (BMPs), Wnt/β-catenin signaling, SMAD-mediated transcription, and insulin-like growth factor (IGF) pathways, thereby being associated with both lineage commitment and terminal maturation of osteoblasts [7–9]. Importantly, osteogenesis is accompanied by dynamic and stage-specific remodeling of miRNA expression, indicating that coordinated temporal regulation is closely linked to orderly progression through the osteogenic differentiation program [10–13].

While numerous miRNAs involved in osteogenesis have been individually characterized, it remains unclear whether MSC populations exhibiting distinct osteogenic capacities, such as DPSCs and PLA-derived MSCs, display intrinsic and lineage-associated miRNA expression programs that persist throughout differentiation. Understanding whether such regulatory landscapes are associated with differentiation trajectories is critical for elucidating how cellular origin relates to cell fate decisions. This knowledge gap is particularly relevant in the context of skeletal biology, where differences in differentiation efficiency may reflect variation in post-transcriptional control rather than disparities in inductive signaling alone.

In this study, we compared the osteogenic differentiation behavior of DPSCs and PLA-derived MSCs under identical in vitro induction conditions and performed longitudinal miRNA profiling across multiple stages of differentiation. By integrating functional mineralization analyses with temporal miRNA expression profiling and targeted validation of selected downstream effectors, we aimed to determine whether distinct miRNA expression landscapes are associated with divergence in osteogenic maturation and to identify post-transcriptional signatures linked to lineage-dependent differentiation outcomes.

## Materials and methods

### Study design and experimental overview

This study was designed to compare osteogenic maturation of mesenchymal stem cells (MSCs) derived from two distinct anatomical sources and to identify microRNA (miRNA) expression programs associated with differential osteogenic behavior. Dental pulp stem cells (DPSCs) and processed lipoaspirate (PLA)-derived MSCs were cultured under identical osteogenic induction conditions and analyzed longitudinally at defined time points (days 0, 7, 14, and 21). Osteogenic differentiation was assessed by extracellular matrix mineralization, while miRNA expression dynamics were characterized by high-throughput small RNA sequencing. Comparative analyses at the mature osteoblast stage (day 21) were performed to identify lineage-associated miRNA signatures, followed by network analysis and targeted validation of selected miRNAs and downstream target genes.

### Cell sources and culture conditions

Deciduous teeth were obtained from six pediatric donors (6–10 years old) following informed consent from legal guardians. Dental pulps were retrieved by endodontic access and immediately transferred to sterile DMEM/F12 medium (Gibco). DPSCs were isolated according to the protocol described by de Mendonça Costa et al. (2010) [14]. Processed lipoaspirate (PLA) cells were isolated from sub-abdominal adipose tissue collected during elective liposuction procedures from six adult donors (24–68 years old), following the protocol established by Zuk et al. (2002) [15].

Both DPSC and PLA cultures were expanded under identical conditions in DMEM/F12 supplemented with 15% fetal bovine serum, 1% penicillin–streptomycin, and 1% MEM non-essential amino acids (Gibco). Cells were maintained at 37 °C in a humidified atmosphere with 5% CO₂, with medium changes every 3 days. Passaging was performed every 4–5 days to maintain subconfluence, and experiments were conducted using cells between passages 3 and 8 to minimize variability unrelated to cell source. All cultures were confirmed to be free of mycoplasma and microbial contamination prior to experimentation.

### Immunophenotyping characterization of MSCs

To confirm mesenchymal identity and exclude endothelial or hematopoietic contamination prior to differentiation, flow cytometric immunophenotyping was performed. Cells were incubated for 30 min at 4 °C with fluorochrome-conjugated antibodies against human CD29, CD73, CD90, CD105, CD31, CD34, and CD45 (BD Biosciences). Isotype-matched controls were included. Data acquisition was performed using a FACSCalibur cytometer (BD Biosciences), and analysis was conducted using CellQuest software. At least 1 × 10⁵ events were recorded per sample.

### In vitro osteogenic differentiation and mineralization assay

For osteogenic induction, DPSCs and PLA-derived MSCs were seeded at approximately 80% confluence and cultured using the StemPro^®^ Osteogenesis Differentiation Kit (Gibco), following the manufacturer’s instructions. This medium contains dexamethasone, β-glycerophosphate, and ascorbic acid as osteoinductive supplements. Media were replaced every 3 days. Cells maintained in standard growth medium served as negative controls.

Extracellular matrix mineralization was evaluated at days 0, 7, 14, and 21. Cultures were washed with phosphate-buffered saline (PBS), fixed in 70% ethanol for 30 min, and stained with Alizarin Red S (0.5 mg/mL; Sigma) for 30 min at room temperature. Bound dye was eluted using 20% methanol and 10% acetic acid, and absorbance was measured at 450 nm using a Tecan Infinite 200 PRO plate reader. Mineralization assays were performed in biological triplicates for each donor and time point.

### RNA isolation and small RNA sequencing

Total RNA was extracted from osteogenically induced and control cultures at days 0, 7, 14, and 21 using TRIzol reagent (Invitrogen), according to the manufacturer’s protocol. RNA concentration and purity were assessed by NanoDrop spectrophotometry (Thermo Scientific), and RNA integrity was evaluated using an Agilent Bioanalyzer. Small RNA fractions were enriched using the PureLink™ miRNA Isolation Kit (Invitrogen). Library preparation was performed using the SOLiD™ Total RNA-Seq Kit, and sequencing was conducted on a SOLiD4 platform to generate 35 bp single-end reads. Biological triplicates were analyzed for each condition and time point.

### miRNA expression profiling and differential expression analysis

Sequencing reads were trimmed and aligned to miRBase v21 using CLC Genomics Workbench v5.1, allowing up to one mismatch. miRNAs with fewer than 20 counts per million (CPM) in at least three samples were excluded from further analysis [16]. Differential expression analyses were performed using the edgeR package (v3.22.4) in R (v3.5.1), with normalization and dispersion estimation conducted according to standard workflows. Comparisons were performed (i) longitudinally within each MSC source (days 7, 14, and 21 versus day 0), and (ii) between DPSC- and PLA-derived cells at day 21. miRNAs with a false discovery rate (FDR) < 0.05 and absolute fold change > 2 were considered significantly differentially expressed.

### miRNA network and pathway analysis using miRNet

To explore functional associations between differentially expressed miRNAs and osteogenesis-related gene networks, miRNA–target interaction and pathway analyses were performed using miRNet (https://www.mirnet.ca/miRNet/) [17]. Analyses were restricted to experimentally validated human miRNA–mRNA interactions. Functional enrichment was performed for skeletal development, extracellular matrix organization, and cell differentiation pathways. Network topology metrics were used to identify highly connected nodes, without inferring direct causal relationships.

### qRT-PCR validation of selected miRNAs and target genes

Selected miRNAs (miR-10a-5p, miR-196a-5p, miR-204-5p, and miR-31-5p) were validated by qRT-PCR in an expanded donor cohort (*n* = 6 per group) at days 0 and 21. miRNA selection was based on differential expression between MSC sources, temporal regulation during osteogenesis, and reported association with osteogenic pathways. cDNA synthesis and miRNA quantification were performed using the miScript system (Qiagen), with RNU6b as endogenous control. For mRNA validation, BMP1 and MMP16 expression were quantified using GoTaq^®^ qPCR Master Mix (Promega). PUM1 and HMBS were used as reference genes. Relative expression was calculated using the ΔΔCt method [17]. Primers are described in Table 1.

**Table 1:** List of primers

BMP1 Fw:	TCCACGTGGGCCTCACA
BMP1 Rev:	ATGAGGGTGCTGCTCTCACTGT
MMP16 Fw:	TCTAGCTATTCTTCGTCGTGAGATGT
MMP16 Rev:	AGTAAGTAATTTGCATTGGGTATCCA
PUM1 Fw:	TGTACTTACGAAGAGTTGCGATGTG
PUM1 Rev:	CCAGGCCAGCGGAAGAT
HMBS Fw:	GGCAATGCGGCTGCAA
HMBS Rev:	GGGTACCCACGCGAATCAC

### Cross-validation with public GEO datasets

To assess the consistency of identified miRNA dynamics with previously published studies, a systematic cross-validation was performed using publicly available GEO datasets. A systematic search of the GEO database was performed using the following MeSH-based query: ((“mesenchymal stem cells” OR MSC OR “adipose-derived stem cells” OR ADSC OR “dental pulp stem cells” OR DPSC) AND (osteogenic OR osteogenesis OR osteoblast) AND (microRNA OR miRNA)) AND “Homo sapiens”[porgn: txid9606]. This search returned 110 files corresponding to 40 distinct GEO datasets. Each dataset was manually curated based on predefined inclusion and exclusion criteria, including cell type, experimental design, temporal resolution, biological context, and type of miRNA profiling. Only three datasets were fully aligned with the objective of investigating temporal miRNA regulation during osteogenic differentiation of human mesenchymal stem cells and were therefore included in the integrative analysis. Inclusion criteria: Human MSCs / DPSCs cultured in vitro; Osteogenic differentiation; Intracellular miRNA profiling; Temporal design (≥ 2 time points). Exclusion criteria: Serum / plasma / exosomal miRNAs; Disease-driven datasets (fracture, diabetes, inflammatory conditions); non-miRNA profiling (circRNA, methylation); non-osteogenic differentiation; Single time point or non-comparable design. The final list of included datasets was: GSE107279, GSE148049, GSE178679.

### Protein–protein interaction network analysis (STRING)

Protein–protein interaction (PPI) analysis was performed to assess whether genes associated with osteogenesis-related miRNAs converge into functionally organized protein networks. Target genes associated with hsa-miR-10a-5p, hsa-miR-204-5p, and hsa-miR-335-5p were compiled based on curated miRNA–target interaction databases, prioritizing experimentally supported and high-confidence predicted interactions. The resulting gene list was analyzed using the STRING database (version 11.5; (https://string-db.org/) [18]) with the organism set to *Homo sapiens*. A medium confidence interaction score threshold (combined score ≥ 0.4) was applied. All available sources of interaction evidence were considered, including experimental data, curated databases, co-expression, text mining, and computational predictions. Disconnected nodes were excluded from network visualization. Network topology parameters, including number of nodes, number of edges, average node degree, and clustering coefficient, were calculated automatically by STRING. Protein–protein interaction enrichment was assessed by comparing the observed number of interactions to that expected for a random network of similar size, with statistical significance reported as the PPI enrichment p-value. Functional enrichment analyses were conducted within STRING using Gene Ontology (biological process, molecular function, and cellular component), KEGG, Reactome, and WikiPathways databases. Enrichment results were considered significant at a false discovery rate (FDR) < 0.05. The STRING analysis was used to identify functional clustering and pathway-level convergence of miRNA-associated targets, without inferring direct regulatory or causal relationships.

### Statistical analysis

Statistical analyses were performed using GraphPad Prism v4.03 and R. Data distribution and sample size guided the use of non-parametric tests. Comparisons between two groups were performed using the Mann–Whitney test. For multi-group comparisons across differentiation time points, Kruskal–Wallis tests followed by Dunn’s post hoc test were applied where appropriate. Results are presented as mean ± standard deviation unless otherwise indicated. Statistical significance was defined as *p* < 0.05. For miRNA sequencing, differential expression analyses were performed using edgeR (v3.22.4) in R 3.5.1 with Benjamini–Hochberg correction. miRNAs with FDR < 0.05 and fold change > 2 were considered significant.

## Results

### DPSCs and PLA-derived MSCs display comparable mesenchymal identity

Dental pulp stem cells (DPSCs) and processed lipoaspirate (PLA)-derived cells exhibited similar fibroblast-like morphology under standard culture conditions. Flow cytometric analysis confirmed that both cell populations fulfilled established mesenchymal stem cell criteria, with more than 90% of cells expressing canonical MSC markers (CD29, CD73, CD90, and CD105), and minimal expression of endothelial (CD31 < 0.5%) and hematopoietic markers (CD34 and CD45 < 1.5%) (Supplementary Table 1). These findings indicate that DPSCs and PLA-derived cells are phenotypically comparable at baseline, enabling a direct comparison of their osteogenic differentiation behavior under identical culture conditions.

**Fig. 1:**
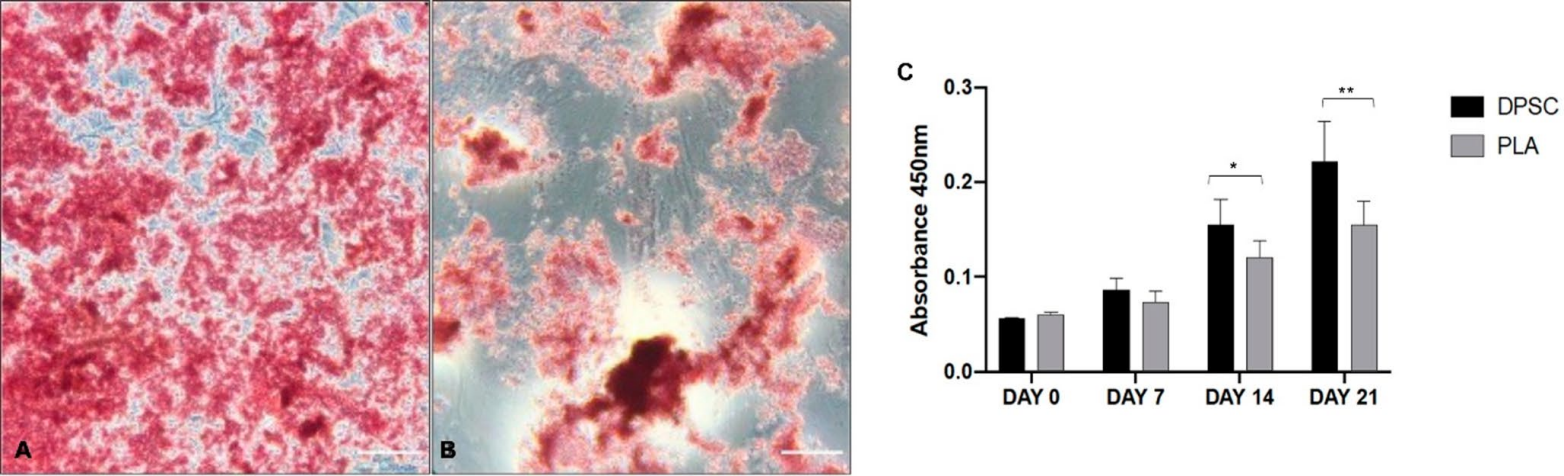
Longitudinal assessment of in vitro osteogenic differentiation. (**A**) Dental pulp stem cells (DPSC) and (**B**) processed lipoaspirate (PLA)-derived MSCs after 21 days of osteogenic induction. Cultures were stained with Alizarin Red S to visualize mineralized extracellular matrix deposition (inverted microscopy, Olympus CKX31; scale bar = 50 μm). (**C**) Quantitative analysis of extracellular matrix mineralization assessed by Alizarin Red S elution at days 0, 7, 14, and 21. Note that DPSCs exhibit significantly higher mineral deposition compared to PLA-derived MSCs specifically at days 14 and 21, indicating a divergence during late-stage maturation. Data are presented as mean ± SD of biological triplicates (*n* = 6 donors per group). Statistical significance was determined by the Kruskal-Wallis test followed by Dunn’s post hoc test (*p < 0.05, **p < 0.01)

Upon osteogenic induction, both DPSCs and PLA-derived MSCs deposited mineralized extracellular matrix over time (Fig. 1A–B). Quantitative Alizarin Red S analysis revealed no significant differences between the two cell types at early stages of differentiation (days 0 and 7). In contrast, DPSCs exhibited significantly higher mineralization at day 14 (*p* = 0.003) and day 21 (*p* < 0.001) compared with PLA-derived MSCs (Fig. 1C). became evident at mid-to-late stages of differentiation, suggesting that divergence between the two cell sources is primarily associated with later phases of osteogenic maturation rather than with early commitment. These results establish a functional difference between the two MSC sources under identical induction conditions and provide the basis for subsequent molecular analyses [5, 19, 20].

### Osteogenic differentiation is accompanied by progressive miRNA remodeling in both MSC sources

**Fig. 2:**
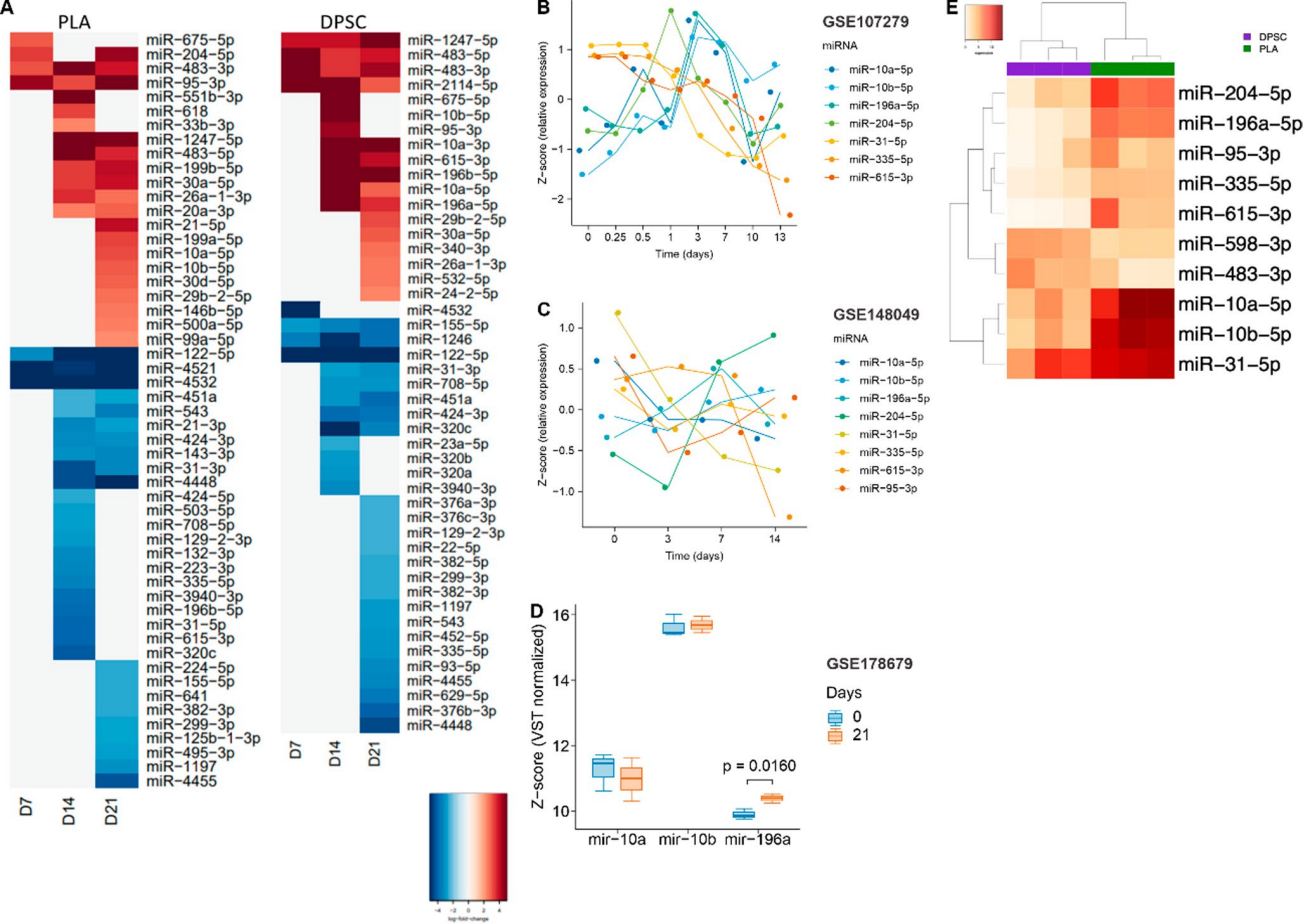
miRNA expression profile during osteogenic differentiation. (**A**) Global miRNA expression dynamics in DPSCs and PLA-derived MSCs during osteogenic differentiation at days 0, 7, 14, and 21. The number of differentially expressed miRNAs (absolute fold change > 2, FDR < 0.05) progressively increases over time in both cell types, reflecting coordinated miRNA remodeling during osteogenesis. Shared and distinct temporal patterns highlight source-dependent differences in miRNA regulation accompanying differentiation. (**B**-**D**) Integration with publicly available GEO datasets (GSE107279 – B; GSE148049 – C; GSE178679 – D) of human MSC osteogenic differentiation revealed consistent temporal downregulation of several miRNAs identified in this study, including miR-10a-5p, miR-10b-5p, miR-196a-5p, miR-204-5p, miR-31-5p, miR-335-5p, miR-615-3p, and miR-95-3p. This cross-validation supports the association of these miRNAs with osteogenic progression across multiple MSC sources and experimental contexts. (**E**) Differentially expressed miRNAs between DPSC- and PLA-derived osteoblasts at day 21 of osteogenic differentiation. Ten miRNAs were identified as lineage-associated, with DPSCs showing reduced expression of multiple miRNAs previously associated with negative regulatory modules in osteogenesis and increased expression of two miRNAs linked to osteogenic signaling. This signature defines a distinct miRNA landscape associated with enhanced osteogenic maturation in DPSCs. FDR < 0.05 and absolute fold change > 2

To characterize molecular dynamics accompanying osteogenic differentiation, miRNA expression profiles were analyzed longitudinally at days 0, 7, 14, and 21 (Fig. 2A). Across all samples, 352 miRNAs were robustly detected after quality filtering. Both DPSC and PLA cultures exhibited progressive changes in miRNA expression over time, consistent with ongoing differentiation. In PLA-derived MSCs, 7 miRNAs were differentially expressed at day 7 relative to day 0, increasing to 33 miRNAs at day 14 and 37 miRNAs at day 21 (absolute fold change > 2, FDR < 0.05). DPSCs showed a similar temporal pattern, with 8, 24, and 39 differentially expressed miRNAs at days 7, 14, and 21, respectively. Upregulated and downregulated miRNAs were observed in both cell types, indicating coordinated remodeling rather than unidirectional shifts in miRNA expression.

Integration with publicly available GEO datasets revealed that several miRNAs, including miR-10a-5p, miR-10b-5p, miR-196a-5p, miR-204-5p, miR-31-5p, miR-335-5p, miR-615-3p, and miR-95-3p, consistently decreased during osteogenic differentiation across multiple human MSC sources (Fig. 2B–D). These miRNAs have been previously associated with inhibitory modulation of osteogenic pathways, in line with their downregulation during differentiation. DPSCs exhibited lower basal expression levels of several of these miRNAs compared with PLA-derived MSCs, indicating differences in their initial miRNA expression landscape.

Direct comparison between DPSC- and PLA-derived cells at the mature osteoblast stage (day 21) identified ten differentially expressed miRNAs (Fig. 2E). Eight miRNAs (miR-10a-5p, miR-10b-5p, miR-196a-5p, miR-204-5p, miR-31-5p, miR-335-5p, miR-615-3p, and miR-95-3p) were expressed at significantly lower levels in DPSCs, whereas two miRNAs (miR-483-3p and miR-598-3p) were upregulated relative to PLA-derived osteoblasts. These results define a lineage-associated miRNA signature present at late stages of osteogenic differentiation, which is associated with enhanced matrix mineralization observed in DPSCs.

### Target de-repression supports functional relevance of selected miRNAs

**Fig. 3:**
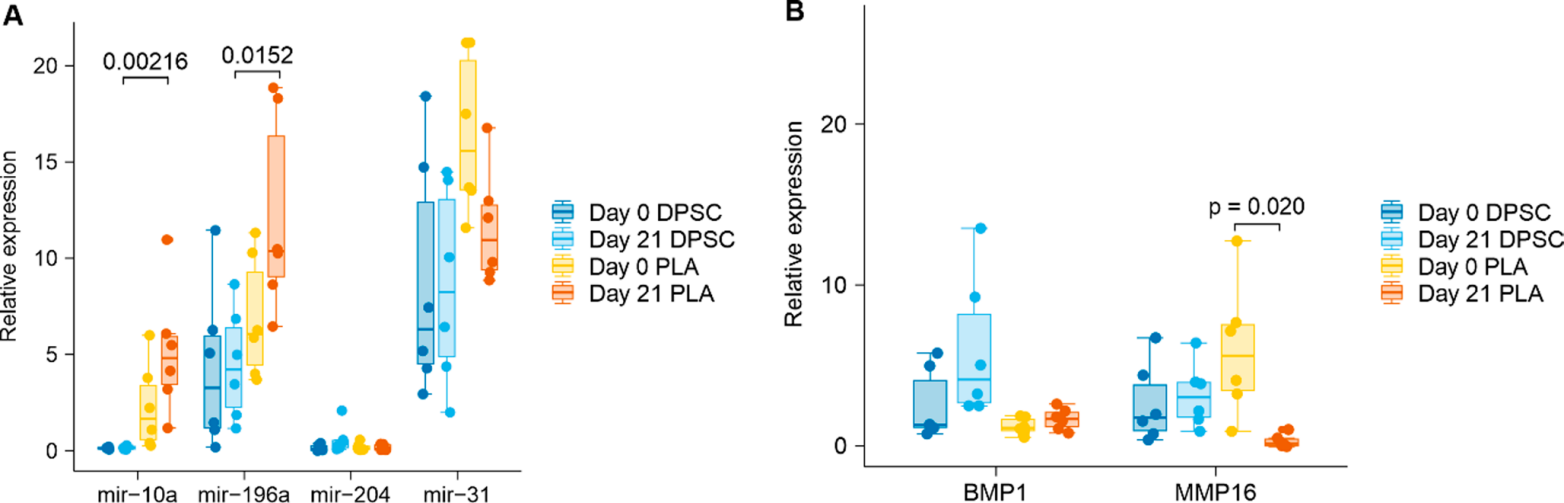
Validation of selected miRNAs and downstream osteogenic effectors. (**A**) qRT-PCR validation of miR-10a-5p, miR-196a-5p, miR-204-5p, and miR-31-5p in an expanded cohort (*n* = 6 per group) at days 0 and 21. DPSCs show significantly lower expression of miR-10a-5p and miR-196a-5p compared to PLAs (Kruskal-Wallis, miR-10a-5p: *p* = 0.00038; miR-196a-5p: *p* = 0.0131). (**B**) mRNA expression analysis of the osteogenesis-related effectors BMP1 and MMP16. The significantly higher expression of these targets in DPSCs is consistent with a model of miRNA-mediated de-repression during late-stage mineralization (BMP1: *p* = 0.00096; MMP16: *p* = 0.0072). Data are shown as mean ± SD

Selected miRNAs were validated by qRT-PCR in an expanded donor cohort. miR-10a-5p and miR-196a-5p exhibited significantly lower expression in DPSCs compared with PLA-derived MSCs at both day 0 and day 21 (*p* < 0.05), confirming sequencing results (Fig. 3A). Although differential expression of miR-204-5p and miR-31-5p was not consistently detected at the miRNA level by qRT-PCR, expression of their predicted downstream targets BMP1 and MMP16 was significantly higher in DPSCs (Fig. 3B). These genes are involved in extracellular matrix processing and remodeling, processes relevant to late-stage osteogenic differentiation. Together, these findings indicate an association between miRNA expression patterns and osteogenesis-related effector genes.

### Network analysis links DPSC-associated miRNAs to skeletal development pathways

**Fig. 4:**
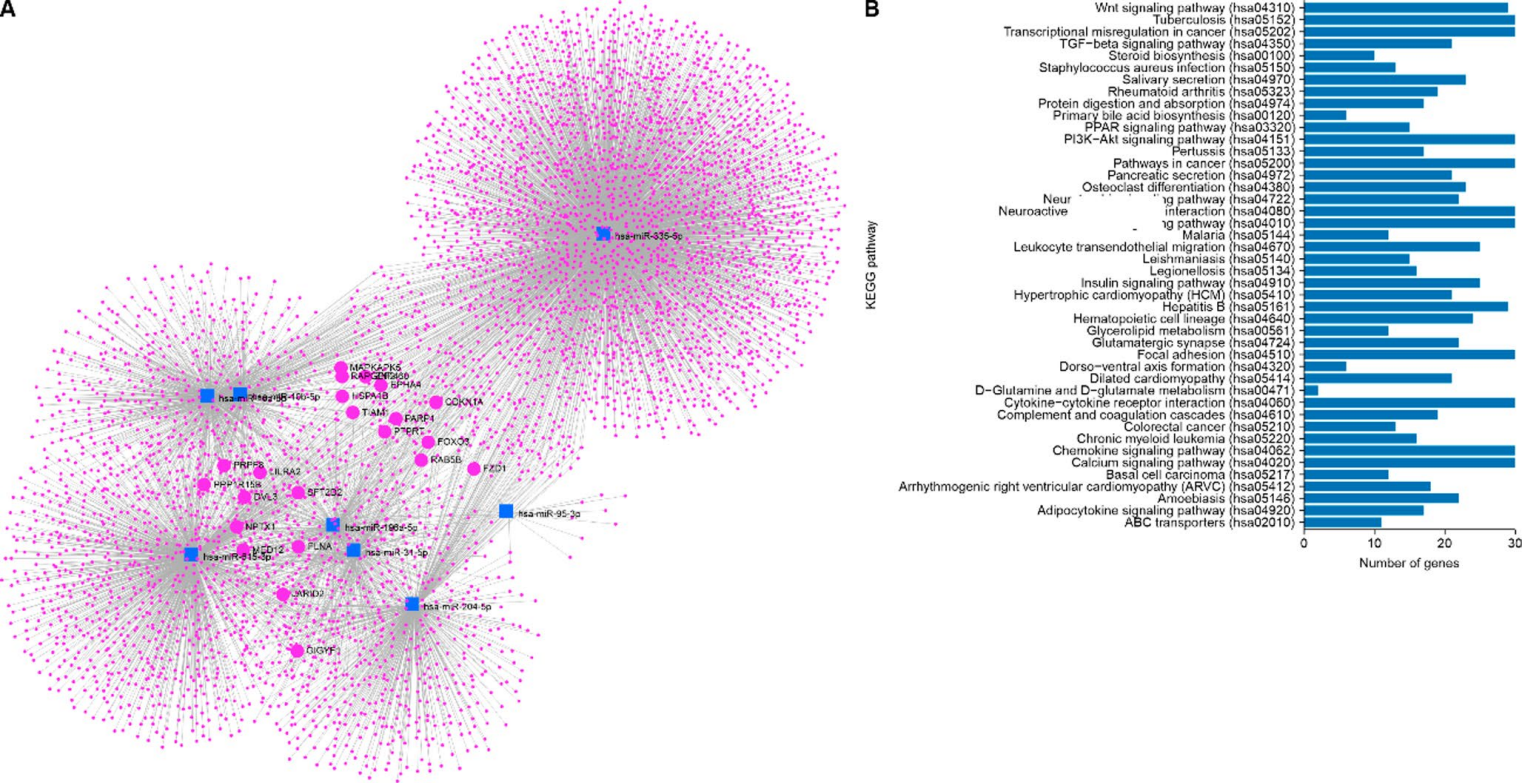
miRNet network analysis of DPSC-associated miRNAs. miRNA–target interaction network generated using the miRNet platform. The network illustrates the potential functional convergence of miRNAs downregulated in DPSCs on gene modules involved in skeletal system development, extracellular matrix organization, and cell differentiation. Node size reflects connectivity (degree), highlighting the coordinated nature of the miRNA regulatory architecture associated with enhanced osteogenic maturation in DPSCs. These results provide a systems-level view of the molecular landscape without implying direct causal relationships

To explore potential functional associations between lineage-associated miRNAs and osteogenesis-related gene networks, miRNA–target interaction and pathway analyses were performed using the miRNet platform (Fig. 4A). Network analysis indicated that miRNAs downregulated in DPSCs are connected to gene networks enriched for skeletal system development, extracellular matrix organization, and cell differentiation (Fig. 4B). These analyses provide an exploratory view of functional convergence of lineage-associated miRNAs on biological processes relevant to osteoblast maturation and matrix remodeling, without implying direct regulatory mechanisms. The integration of multiple miRNAs within shared network structures suggests that enhanced osteogenic performance in DPSCs is associated with coordinated patterns of gene expression rather than isolated molecular effects.

To further examine whether targets associated with osteogenesis-related miRNAs cluster at the protein interaction level, a protein–protein interaction network was generated using the STRING database for genes associated with hsa-miR-10a-5p, hsa-miR-204-5p, and hsa-miR-335-5p. The resulting network exhibited significantly more interactions than expected by chance (PPI enrichment *p* < 1.0 × 10⁻¹⁶). Functional enrichment analysis showed overrepresentation of pathways related to transforming growth factor beta signaling, SMAD phosphorylation, protein kinase activity, and bone remodeling-associated processes. These results highlight coordinated clustering of proteins involved in signaling and matrix-related pathways relevant to osteogenic maturation, without implying direct regulatory relationships (Supplementary Fig. 1).

## Discussion

Mesenchymal stem cells derived from distinct anatomical sources are increasingly recognized as biologically heterogeneous populations, despite sharing canonical mesenchymal phenotypic criteria [1, 2, 7, 13]. This heterogeneity reflects intrinsic molecular differences that influence lineage commitment, differentiation progression, and terminal maturation [3, 4, 6, 21]. In the context of skeletal biology, such variability represents a fundamental challenge, as osteogenic outcomes cannot be fully predicted based on phenotypic identity or inductive conditions alone [9, 10, 14]. In this study, we show that dental pulp stem cells (DPSCs) exhibit increased late-stage extracellular matrix mineralization compared with processed lipoaspirate (PLA)-derived MSCs under identical induction conditions, and that this divergence is associated with a distinct and temporally coordinated miRNA expression landscape.

Consistent with previous reports [4, 10, 20, 22, 23], DPSCs exhibited significantly greater extracellular matrix mineralization at mid-to-late stages of osteogenic differentiation. The absence of differences at early time-points indicates that both cell populations are capable of initiating osteogenic commitment. The emergence of divergence during later stages suggests that regulatory differences are primarily associated with osteoblast maturation and matrix mineralization rather than with initial lineage entry. This distinction indicates that intrinsic regulatory features, rather than differences in inductive signaling, are associated with variation in osteogenic efficiency [9, 14, 24].

Longitudinal profiling revealed progressive remodeling of miRNA expression in both MSC sources during osteogenic differentiation, consistent with prior studies demonstrating that osteogenesis is accompanied by dynamic and stage-specific miRNA regulation [8, 11, 25, 26]. Several miRNAs identified in this study, including miR-10a/b, miR-196a, miR-204, miR-31, miR-335, miR-615, and miR-95, have been previously implicated in negative modulation of osteogenic pathways through effects on transcriptional activity, growth factor signaling, and extracellular matrix organization [7, 8, 11, 27–31]. The conserved temporal downregulation of these miRNAs across independent MSC datasets further supports their association with osteogenic progression [25, 32].

A central observation of this work is that DPSCs exhibit reduced basal and late-stage expression of multiple miRNAs associated with inhibitory regulatory modules in osteogenesis. This pattern is consistent with the presence of a pre-existing post-transcriptional expression landscape associated with fewer miRNA-mediated constraints during osteogenic maturation. Such intrinsic expression profiles may contribute to differences in the efficiency with which osteogenic gene programs are activated during differentiation and provide a conceptual framework that is concordant with the increased mineralization capacity observed in DPSCs [14, 15]. Because proliferation and viability were not directly quantified during differentiation, we cannot exclude the possibility that differences in cell number or survival contributed to the magnitude of matrix mineralization observed between DPSC and PLA-derived cultures. Future studies incorporating parallel assessment of cell growth kinetics and viability will be required to further disentangle regulatory effects from cell density–related influences on extracellular matrix deposition.

Comparative analysis at the mature osteoblast stage identified a panel of ten miRNAs that distinguish DPSC- and PLA-derived osteoblasts. These miRNAs do not appear to act as isolated regulators but instead converge on shared biological processes related to skeletal development, extracellular matrix remodeling, and cell differentiation, as revealed by network-based analyses [8, 14, 15, 17, 33]. This convergence supports a model in which coordinated modulation of miRNA networks is associated with differentiation trajectories, rather than individual miRNAs functioning as dominant molecular switches [11, 15].

Associative links between the identified miRNA landscape and osteogenic maturation were further supported by targeted validation analyses. Although differential expression was not uniformly confirmed for all miRNAs at the transcript level, reduced expression of miR-10a-5p and miR-196a-5p in DPSCs was consistently observed, accompanied by increased expression of downstream osteogenesis-associated effectors, including BMP1 and MMP16. These molecules participate in extracellular matrix processing and remodeling, processes relevant to late-stage osteoblast maturation and mineral deposition [14, 15, 34]. While these findings support an association between miRNA expression patterns and downstream effector pathways, functional manipulation of BMP1 and MMP16 will be required to determine whether modulation of these targets is sufficient to enhance osteogenic differentiation in PLA-derived MSCs and to recapitulate aspects of the DPSC phenotype [8, 16, 35].

Several limitations should be considered when interpreting these findings. Because donor age differed between the DPSC and PLA-derived MSC populations, age represents a potential confounding variable in the interpretation of both osteogenic behavior and miRNA expression profiles. Although the identified miRNA signatures were consistent across biological replicates and concordant with previously reported osteogenesis-associated miRNAs, age-related influences on differentiation efficiency and post-transcriptional regulation cannot be excluded [3, 4, 10, 20]. Therefore, the observed differences are interpreted as being associated with lineage-dependent regulatory programs in the context of potential age-related effects, rather than being attributable to cellular origin alone. Moreover, because this study is based on longitudinal expression profiling and target association analyses, the identified miRNA–gene relationships should be interpreted as associative rather than causal. Direct functional validation using miRNA mimics or inhibitors will be necessary to determine whether individual miRNAs are sufficient to modulate osteogenic capacity [11, 25]. The integration of longitudinal profiling, network analyses, and target validation provides a structured framework for future mechanistic investigation of post-transcriptional regulation during osteogenic differentiation [8, 14, 18, 19, 36].

In addition to intracellular regulatory programs, extracellular vesicle–mediated transfer of miRNAs has been increasingly recognized as an important mechanism of intercellular communication in bone biology [37]. Future studies should therefore examine whether lineage-associated miRNA signatures identified at the cellular level are also reflected in the cargo of extracellular vesicles derived from DPSCs and PLA-derived MSCs, and whether such vesicle-associated miRNAs contribute to paracrine modulation of osteogenic differentiation.

Beyond these considerations, the present findings have broader implications for understanding mesenchymal lineage specification. The identification of lineage-associated miRNA expression landscapes highlights the importance of intrinsic post-transcriptional programs in shaping differentiation trajectories and contributes to a growing conceptual framework in which stem cell fate is influenced by coordinated regulatory networks rather than isolated signaling pathways [38].

In summary, this study identifies a distinct and temporally coordinated miRNA expression architecture in DPSCs that is associated with increased late-stage extracellular matrix mineralization. By linking differentiation outcomes to intrinsic post-transcriptional expression patterns, these findings provide insight into how cellular origin is associated with lineage-specific differentiation trajectories and underscore the association between miRNA-mediated regulatory landscapes and osteogenic differentiation behavior [8, 14, 15].

## Conclusions

This study demonstrates that dental pulp–derived mesenchymal stem cells exhibit increased late-stage extracellular matrix mineralization compared with processed lipoaspirate-derived MSCs under identical inductive conditions. This divergence emerges predominantly at mid-to-late stages of differentiation and is associated with differences in intrinsic regulatory programs rather than variation in mesenchymal identity or early lineage commitment. We identify a lineage-associated miRNA expression signature distinguishing osteogenic outcomes between these MSC sources, with DPSCs characterized by reduced expression of miRNAs previously linked to inhibitory post-transcriptional regulation of osteogenesis. Network-level and target-based analyses indicate that these miRNA patterns converge on gene modules involved in skeletal development and extracellular matrix remodeling. Together, these findings indicate that intrinsic, lineage-associated miRNA expression landscapes are associated with distinct late-stage mineralization trajectories, in the context of potential donor-related influences such as age in human MSCs and provide insight into how cellular origin relates to differentiation behavior during osteogenesis.

## Supplementary Information

Below is the link to the electronic supplementary material.

Supplementary Material 1

Supplementary Material 2

## Data Availability

The data supporting the findings of this original article is publicly available and shared in the Figshare repository: 10.6084/m9.figshare.30687080.
