# Supplementary material for: Intrinsic microRNA regulatory programs define lineage-specific differentiation in human mesenchymal stem cells of different origin – dental pulp- and fat tissue-derived
Source: Stem Cell Rev Rep. 2026 Mar 22;22(4):1946–57. doi: 10.1007/s12015-026-11107-7 (PMC13099662; doi:10.1007/s12015-026-11107-7)
Supplement: Supplementary file 2 — Supplementary Material 2 [file 12015_2026_11107_MOESM2_ESM.docx]

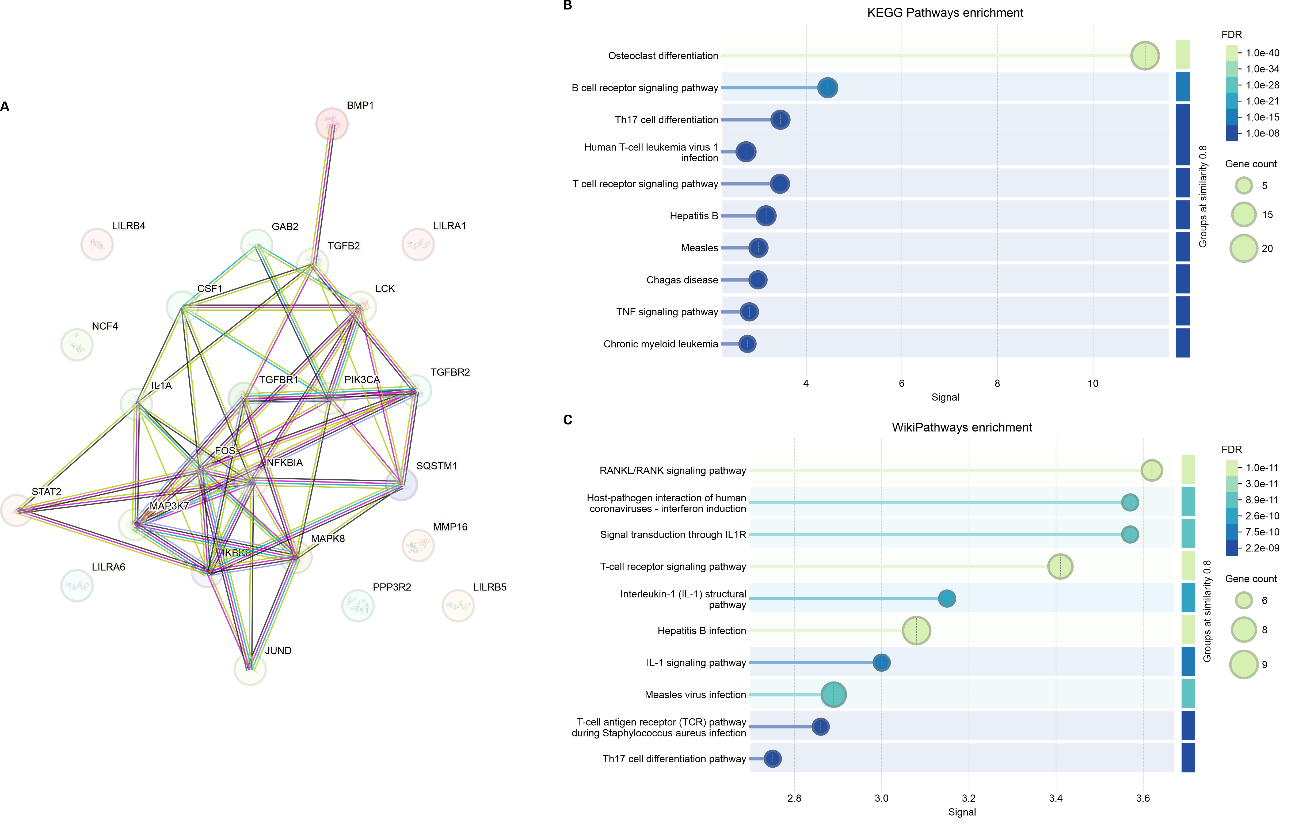


**Supplementary Figure 1. Protein–protein interaction network of miRNA-associated osteogenic targets.** Network generated via the STRING database (version 11.5) for genes associated with the DPSC miRNA signature (miR-10a-5p, miR-204-5p, and miR-335-5p). The analysis revealed a significant enrichment of interactions compared to a random background (PPI enrichment p < 1.0x10^-16^), with clusters involving TGF-β signaling, SMAD phosphorylation, and bone remodeling pathways. Disconnected nodes were excluded for clarity. This protein-level clustering supports the functional relevance of the identified post-transcriptional signature.
